# Supplementary material for: Colonization of the Gastrointestinal Tract of Chicks with Different Bacterial Microbiota Profiles
Source: Animals (Basel). 2023 Aug 15;13(16):2633. doi: 10.3390/ani13162633 (PMC10451890; doi:10.3390/ani13162633)
Supplement: Supplementary file 1 [file animals-13-02633-s001.zip › animals-2486698-supplementary.pdf]

Supplementary Materials

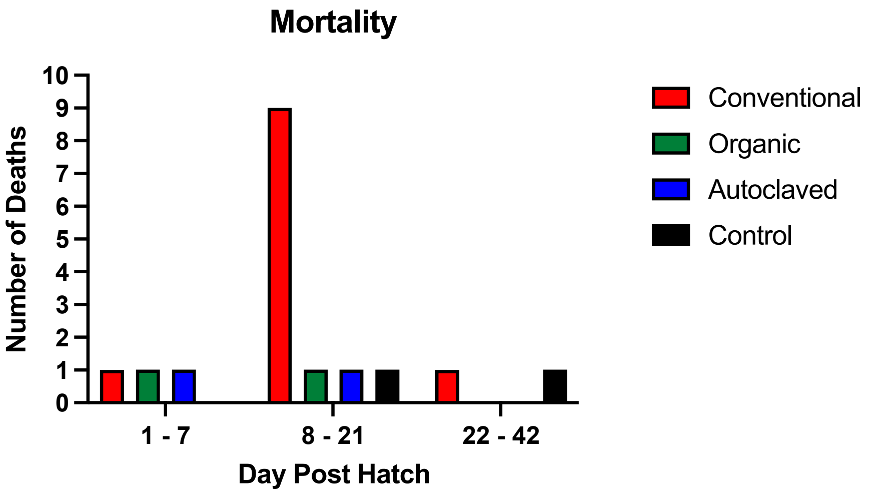

Figure S1. Mortality. Number of deaths measured in each treatment group (n = 75 birds/group), inoculated or non-inoculated with caecal microbiota transplant. Records were recorded daily during the whole trial.

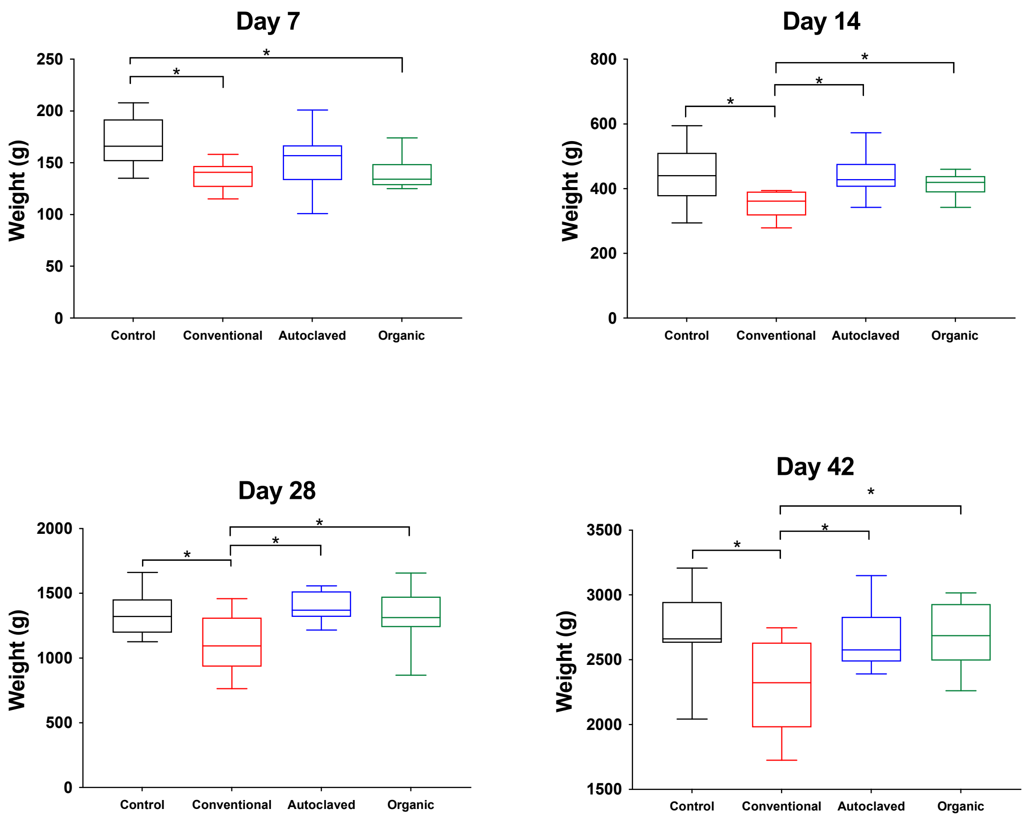

Figure S2. Body weight. Box plot showing the total body weight values on days 7, 14, 28 and 42 of chicks receiving different microbiota profiles. Body weight was calculated separately for each sampling day of the experiment for each treatment group. Bars represent mean and SD. Results were considered statistically significant if $p \leq 0.05$.

Table S1. Primers for RT-qPCR.

Primer name	Sequence (5′ to 3′)	Reference
β-actine F	CAACACAGTGCTGTCTGGTGGTA	[69]
β-actine R	ATCGTACTCCTGCTTGCTGATCC	
GAPDH F	GGTGGTGCTAAGCGTGTTAT	[70]
GAPDH R	ACCTCTGTCATCTCTCCACA	
IL-4 F	AACATGCGTCAGCTCCTGAAT	[71]
IL-4 R	TCTGCTAGGAACTTCTCCATTGAA	
IL-6 F	CGTGTGCGAGAACAGCATGGAGA	[72]
IL-6 R	TCAGGCATTTCTCCTCGTCGAAGC	
IL-10 F	CGGGAGCTGAGGGTGAA	[73]
IL-10 R	GTGAAGAAGCGGTGACAGC	
IL-12 F	AGACTCCAATGGGCAAATGA	[70]
IL-12 R	CTCTTCGGCAAATGGACAGT	
